# Aquatic community structure as sentinel of recent environmental changes unraveled from lake sedimentary records from the Atacama Desert, Chile

**DOI:** 10.1371/journal.pone.0229453

**Published:** 2020-02-21

**Authors:** Adriana Aránguiz-Acuña, José A. Luque, Héctor Pizarro, Mauricio Cerda, Inger Heine-Fuster, Jorge Valdés, Emma Fernández-Galego, Volker Wennrich

**Affiliations:** 1 Centro de Investigación Tecnológica del Agua en el Desierto (CEITSAZA-UCN), Antofagasta, Chile; 2 Departamento de Ciencias Geológicas, Universidad Católica del Norte, Antofagasta, Chile; 3 Laboratório de Biogeoquimica de Ambientes Aquáticos Universidade Federal Fluminense (PPBMAC—UFF), Rio de Janeiro, Brazil; 4 Centro de Investigación e Innovación para el Cambio Climático (CiiCC), UST, Santiago, Chile; 5 Instituto Alexander von Humboldt, Universidad de Antofagasta, Antofagasta, Chile; 6 Institute of Geology & Mineralogy, University of Cologne, Cologne, Germany; University of Utah, UNITED STATES

## Abstract

The Atacama Desert (21–26°S) is currently one of the driest places on Earth and metal(loid)s are of special concern for this region, which hosts the largest-known porphyry copper deposits produced in Chile. Evidence of past environmental conditions is commonly preserved in natural archives, such as lacustrine sediments. Sediment records obtained from Inca Coya Lake (22°20’S-68°35’W, 2534 m.a.s.l.), a small lake located in the Atacama Desert, reflected the evolution of regional mining activity during the 20^th^ century and sedimentation associated with decadal climate variability. We studied the aquatic community structure changes recorded in sediment records from Inca Coya Lake. By analysis of magnetic properties (susceptibility, hysteresis curves and Curie temperatures), grain size and geochemical composition of the sediments, we identified environmental periods and changes in the community of benthic and planktonic organisms (diatoms and diapausing egg bank). We identified three detrital episodes that we interpret as dry/wet phases during the last 90 years associated with the increase of flash flood events promoting hypoxia oscillations; anthropogenic (mining activity) signals were also identified. Invertebrate community structure (primary consumers) reflected the metal exposure, measured as changes in assemblage composition through species turnover. Diatom community composition was best associated with variables related to wetter/drier alternation and consequent changes in oxygen availability. Bioindicators analyzed (diatoms, diapausing egg bank and invertebrate community) demonstrated to be excellent indicators of the bioavailability of compounds in the aquatic ecosystem of Inca Coya Lake, allowing the environmental impact assessment of the water resources due to flash floods and mining activity in the driest desert of the world.

## Introduction

Anthropogenic metal contamination can be an intense stressor on ecosystems and driver of biological population evolution in polluted environments [1, 2]. Activities related to worldwide metal mining such as metal extraction and smelting complexes have generated dramatic consequences in biological communities of aquatic systems [3, 4]. These environmental metal concentrations could result in the alteration of the long-term structure and diversity of aquatic communities, which may be studied by a paleoecotoxicological approach [5].

Evidence of past environmental conditions is commonly preserved in natural archives such as lacustrine sediment records, which inform about the mechanisms of transport and accumulation of important geochemical and fossil archives. Some populations are sensitive to environmental variations, which may be proportional to changes in fossil abundance, evolutionary changes in target populations or species replacement. A paleoecological approach including different trophic groups as paleoindicators will contribute to a better understanding of what environmental variables have affected lake systems and how these variables have impacted the structure of ecological communities. This is because (1) not all species are equally sensitive to all environmental pressures and (2) emerging community attributes cannot be assessed at the level of single species. Ostracods, gastropods, chironomids and phytoplankton, mainly diatoms, are common in both extant desert wetlands and lake deposits [6, 7]. Diatoms are undoubtedly the most important group of algae for paleolimnological studies; diatom-based assessment methods may compare well with methods using other biological quality elements [8]. Zooplankton populations, specifically the herbivores cladocerans and rotifers, are also used frequently in bioassays and as bioindicators of water quality to detect anthropogenic contamination because of their sensitivity to different toxicants and their important ecosystem role. Nevertheless, the structure of zooplankton diapausing egg banks has been little explored as paleolimnological indicator. Diapausing egg banks provide an excellent system for reconstructing conditions experienced before and during diapause, because layered deposits retain both diapausing eggs and traces of the chemical and biological conditions present in the water column at the time of deposition [9]. These records allow tracking historical environmental changes by the ecological and evolutionary responses of populations and past zooplankton communities to those conditions [4, 10–13]. Studies have assessed experimentally the effects of metal(loid)s, among other toxicants, on the life cycle of rotifers [14, 15], showing that specific life history traits associated with diapausing egg production and egg bank hatchability are the most sensitive endpoints to assess metals toxicity in rotifers, and therefore temporal changes in the structure of diapausing egg banks may be associated with changes in the metal(loid) concentrations in the environments.

The Atacama Desert (21–26°S) is currently one of the driest places on Earth [16, 17]. Hyperaridity in the region is attributed to the blocking influence of the southeast Pacific Subtropical High, cold-water upwelling and the rain-shadow effect of the Andes [18]. Associated with the Central Depression and the Coast Range Below 2300 m.a.s.l., a, is a zone of extreme hyperaridity with the mean annual precipitation (MAP) <2 mm per year [19, 20], which is maintained by the high evaporation potential in most areas of the Atacama Desert [19]. Inorganic contaminants such as metals and metalloids are of special concern for northern Chile, which hosts the largest known porphyry copper deposits [21] and ores and copper concentrates produced in the region, which are sources of Mo, As, Re, and to a lesser extent Zn [22]. This great copper exploitation is the main support of economic growth of the country and Antofagasta is the leading region of Chilean copper production, with 2.88 million tons of fine copper, which represent 52% of national production [23]. Large porphyry copper deposits (e.g. Chuquicamata, Escondida, Radomiro Tomic, etc) are located in the Antofagasta Region, in the central Atacama Desert. As a consequence, the mining industry has been extensively developed since the XIX century in the region [24, 25], with a subsequent increase in pollution related to this activity (e.g. [22, 26]).

Recently, Cerda et al. [27] studied a geochemical record and geochronology of a sediment core drilled from Inca Coya Lake (22°20’S-68°35’W, 2534 m.a.s.l.), a small lake located at the eastern margin of the Atacama Desert. Their results clearly reflect the evolution of regional mining activity during the 20^th^ century and sedimentation variability associated with decadal climate variability driven by El Nino-Southern Oscillation–ENSO. Nevertheless, this study is not aim to show the consequences of both factors on the biota of the lake. In previous experimental studies driven in laboratory conditions, we have shown that diapause strategies in rotifer species isolated from Inca Coya Lake are dependent on metal(oid)s exposure and that the pattern of eggs production may modulate the community structure [28]. Toxicant-stressed communities or species could evolve into resistant forms and/or be replaced by pollution-tolerant invaders [29]. These antecedents allow us hypothesize that different levels of exposure to metal(loid)s over time could be reflected in the presence in sediment records of organisms with different degrees of sensitivity. Additionally, ENSO has resulted in anomalous rainfalls in the Atacama Desert producing debris flow along a latitudinal range [30, 31]. ENSO occurrence and magnitude significantly have affected the flood regimes in the Atacama Desert [32]. On recent periods, the 1997–98 ENSO, one of the strongest and best-developed events of the 20th century, produced widespread flooding occurring across the western Andean region [33]. The extremely arid conditions and the isolation of desert lagoons such as Inca Coya Lake make such sites highly vulnerable to increasing and sustained anthropogenic disturbance and to the rapid climate changes promoting stronger rainfall events modulated by ENSO and ENSO-like conditions impacting from arid to semiarid region [32], which place them in the focus of conservation studies for aquatic communities [28].

Here, we used a novel combination of a paleolimnological reconstruction, geochemical analysis, and magnetic properties analysis to identify variation in the aquatic community structure in two sediment cores from Inca Coya Lake associated with indicators of environmental drivers. Our aims were (1) correlate natural (dry/wet phases) and anthropogenic (mining activity) variability factors with the community structure from sediment records, (2) assess the importance of recent floods events on lacustrine systems from arid zones and (3) asses the long-term exposure to metal(loid)s as an evolutionary force on the aquatic community from a Desert lake.

## Materials and methods

### Study site and sampling

**Fig 1:**
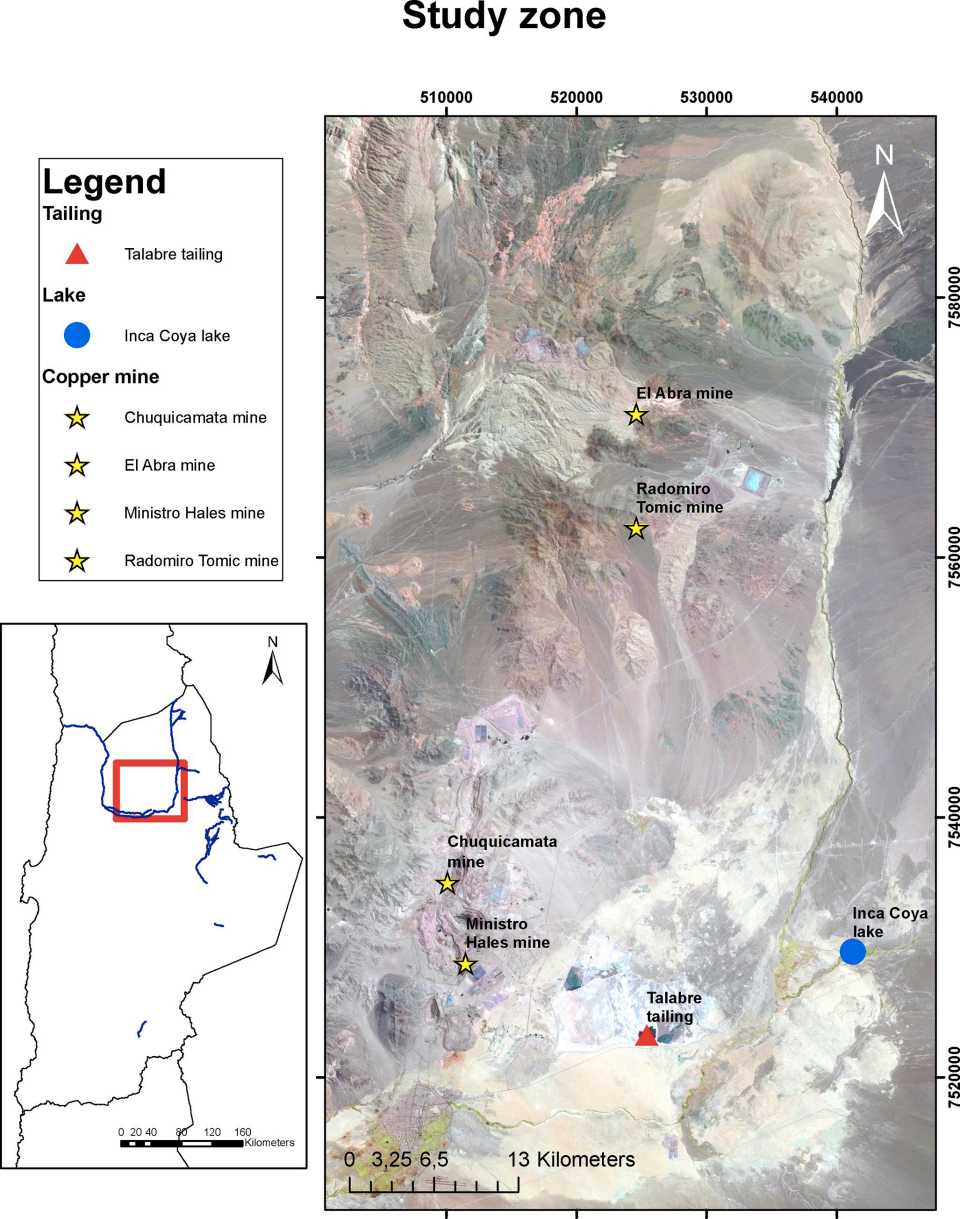
The study site location. Inca Coya Lake (22°20’S-68°35’W), in the context of different mining activities in the zone (DATUM WGS 1984 UTM Zone 19S).

Inca Coya Lake is a small lake with a surface area of 500 m^2^ and a maximum depth of 18 m located near the village of Chiu-Chiu in the Antofagasta Region, Chile (Fig 1). The hyperarid conditions of the Atacama Desert fluctuated in the late Pleistocene and Holocene time-scales. During glacial periods Pacific frontal systems migrated 150–200 km further north than during interglacial periods [34–37], creating wetter conditions in southern areas (>23°S) that are hyperarid today [16, 38, 39]. In contrast, during the last deglaciation beginning at 17 kyr, the Andean foothills experienced significant increases in summer rainfall producing more runoff on piedmont fans located at 19°S [40–43]. Inca Coya Lake is a karstic sinkhole, developed during the Quaternary period by dissolution of calcareous layers of the El Loa Formation. This formation is constituted by homogeneous deposits known as “coba” and a superior sequence of thick materials (sandstones, gaps and conglomerates), which highest section mostly corresponds to lacustrine calcareous sediments known as limestones. The sinkhole is formed by dissolving the calcareous rocks in water, either superficial or underground, saturated with carbon dioxide [44]. Although the lake is not within an area protected by the Chilean State, Chiu-Chiu village and Inca-Coya Lake correspond to Likan Antai territory, and therefore the authorization to access to the lake and develop the fieldwork were previously requested of Oscar Sarapura, President of the Chiu-Chiu community.

Two sediments cores were obtained from the deepest point (18 m) of Inca Coya Lake using a 5 cm diameter gravity core (Wildco, K-B Corer 20”). Sediment cores were collected at the deepest part of the basin where maximum sedimentation rates are assumed. Bathymetric mapping of Inca Coya Lake was previously performed by Cerda et al. [27], identifying the most appropriate site for sediment coring. A first core (CORE1 hereafter), 470 mm long, was retrieved in July 2013. A second shorter core (193 mm) (CORE2 thereafter) was obtained in March 2015 from the same sampling point with the same sampling equipment. To prevent sediment disturbance, cores were kept in their PVC casings and transported carefully in a vertical position and stored frost-free. In the laboratory the cores were split lengthwise, photographed, and one half of each core was sectioned into 1-mm intervals for CORE1 and into 5-mm intervals for CORE2.

Physical-chemical parameters of the water column (temperature, salinity, electrical conductivity, ORP, dissolved oxygen and pH) were measured *in situ* with a multi-parameter device YSI 6600 Water Quality Monitor down to 11 m depth. Large volume sediment samples were obtained with a Lenz bottom sampler (modified version of the Ekman-Birge model), which is a precise quantitative sampler equipped with dividing sheets to separate the sample into 5 layers of 20 mm thickness. These layers can be removed separately for individual examination.

### Grain sizes and magnetic properties

Granulometry from CORE1 was analyzed using a standard sieve of 2-mm mesh and Cilas^®^1180 Laser Particle Analyzer [45] at the Coastal Environments and Marine Biology Program Laboratory (PBMAC-UFF, Fluminense, Brazil). For grain size analysis, samples were dried, homogenized, and carbonates and organic matter were removed by adding aliquotes of HCl_conc_ and H_2_O_2_ (30%) solution until reactions ceased [46]. Dispersion was performed with sodium hexametaphosphate (40 g·L^-1^) followed by sonication (35 W for 2 min). Cilas^®^ analysis was performed on 0.2 g of samples; particle size range spanned from 0.04 to 2500 μm.

A total of 87 samples (ca. 10 g each) were obtained for magnetic analyses from sediment CORE 1. Samples were collected at 0.5 cm intervals down along the stratigraphic profile. To constrain the effects of environmental processes on magnetic minerals present in the sediments (e.g. [47]), samples were ground and homogenized (<0.1 mm) in an agate mortar, were mounted in a Teflon sample holder for magnetic measurements and covered, where each sample was ca 2 mg [48]. These magnetic samples were subjected to hysteresis experiments at room temperature and a pressure of 1 atm using a MicroMag AGM 2900–2 alternating gradient force magnetometer (Princeton Measurements Corp., Princeton, NJ, USA). Magnetic hysteresis parameters were calculated using PmagPy routines [48, 49]).

Four magnetic sub-samples were used to determine Curie temperatures via thermomagnetic experiments using a CS4 MFK1 Kappabridge (AGICO). Powdered samples were heated from room temperature to 700°C (and then cooled to room temperature) under a weak magnetic field (200 A/m) and 1 atm pressure in a non-inert (air) atmosphere. To calculate the Curie points we use a normalized gradient of thermomagnetic curves $grad=(K_{T_{n+1}}-K_{T_{n}})/(T_{n+1}-T_{n})$, where K_T_ is the bulk magnetic susceptibility measured at a given temperature and T is the measurement temperature [48].

### Dating and chronological correlation between cores

Nine sediment samples of CORE1 were dried, powdered and sealed in plastic flasks for ^210^Pb dating (non-destructive analyses) at LARAMG Laboratory/Rio de Janeiro State University-Brazil. The dating method employed the radionuclides ^210^Pb and ^226^Ra from the ^238^U natural series by a high-resolution gamma spectrometry, using an extended energy range co-axial hyperpure germanium (HPGe) detector (model GX5021 –Canberra). Equipment relative efficiency is 50% with a resolution of 2.1 keV (FWHM) at the ^60^Co (1.33 MeV) energy peak. This detector was installed inside a very low background lead shielding of average thickness of 15 cm, internally coated with pure Cu. A detector efficiency curve for the sediment samples was calculated using a liquid solution containing a cocktail of radionuclides—NIST (serial number HV951). The cocktail used in this study included the radionuclides ^133^Ba, ^57^Co, ^139^Ce, ^85^Sr, ^137^Cs, ^54^Mn, ^88^Y, and ^65^Zn, which enclose the energy range of interest. The total counting time for each sample was 24h. The geochronological model used in this work was the Constant Rate of Supply (CRS), where the initial ^210^Pb concentration in the sediment is constant and the influx rate of sediment is variable [50].

Chronological control of the sediment record of CORE2 was performed using the geolimnological correlation between CORE1 and CORE2. We compared the grey-scale profiles and a suite of geochemical proxies from the both sediment cores (mainly metal(loid)s such as As, Cu and Zn). The image analysis of lacustrine sediments is an useful method in paleolimnology and paleoenvironmental reconstructions, and it constitutes an excellent tool for the correlation of several sediment cores. The advantage of this method is the high-resolution capacity and therefore the close spacing of the data points. Image analysis has shown to be a useful approach in many contexts, and grey-scale variation data has been used successfully to correlate cores [51].

### Geochemical analysis

Geochemical analyses of 466 samples of CORE1 were shown in Cerda et al. [27]. Element data of CORE 2 were non-destructively analyzed at 100-micron resolution on the untreated archive half using an ITRAX XRF core scanner (Cox Analytical Systems, Sweden) equipped with a Cr X-ray tube set to 30kV and 55 mA. A measuring time of 50 s per point was chosen for data acquisition.

Zonation of the geochemical element data was conducted using the constrained incremental sum of squares (CONISS) function in TILIA v.1.7.16.

### Biological proxies

For diatom analyses, 19 sediment samples of CORE2 were treated by adding hydrogen peroxide (30%) to 5 mg of freeze-dried material and heating in a water bath to degrade the organic material and retain the diatoms. The diatom community was identified using a Zeiss Axioplan microscope, a minimum of 400 valves in each sediment sample were counted, based on standard diatom identification literature [52]. The abundance of each taxonomic group per layer was estimated. Based on geochemical and diatoms composition, zonation was obtained by CONISS using the broken stick method to determine the significant zones [53].

Invertebrates were separated, identified and counted from the five sediment samples obtained with the bottom sampler. Fully benthic invertebrates (benthic) and zooplankton diapausing eggs forming egg banks in the sediment (planktonic), were analyzed separately.

Detrended correspondence analysis (DCA) [54] with detrending by segments was applied to the diatom percentage data to explore the temporal patterns of species changes [55]. A DCA axis gradient length of 1.65 standard deviations (SD) was obtained, indicating that RDA (redundancy analyses) was appropriate to explore the relationships between the diatom community and the environmental variables [56]. Permutation tests were used to test for the significance of each environmental variable in the RDA, and only those variables with p < 0.05 under 999 permutations were accepted for model selection [56]. Forward selection was used to identify environmental variables in the minimum adequate model that exerted significant influence on the biological data, with Akaike’s Information Criterion (AIC) used as the selection criterion [57].

Once the reduced model was obtained, co-linearity between environmental variables was evaluated by the analysis of Variance Inflation factors (VIF) <10. The method of variance partitioning was applied to analyze the independent and interactive role of the selected variables in affecting biological changes with the application of RDA [54, 58].

DCA was also performed to evaluate turnover of benthic and planktonic communities through time. Community changes were calculated as the Euclidean distance between adjacent samples, estimated using the first four DCA axes [59–61]. Rates of ecological change (RoC) were calculated as the dissimilarity between two contiguous samples (time-slices), derived by dividing the distance by the time elapsed between them [60]. The mean sedimentation rate estimated for this time interval in CORE1 was used to calculate the RoC, which was 0.19 cm·yr^-1^ [27].

All statistical analyses were performed in the R program (version 2.2.1) using the vegan [62] and PaleoMas [63] packages.

## Results

Standard physical-chemical parameters of the water column of Inca Coya Lake are shown in Table 1. Based on these parameters and the chlorophyll concentration in water column, Inca Coya Lake is characterized as a holomictic and alkaline [64], as well as an oligotrophic water body, according to the trophic classification system of OECD [65] respectively.

**Table 1:** Water column parameters.

Depth (m)	Salinity (g L^-1^)	pH	Chl *a* (μg L^-1^)	O_2_ (mg L^-1^)	Conductivity (μS cm^-1^)	ORP (mV)
**0**	5.28	9.26	1.58	6.27	7463	39.4
**1**	5.29	9.25	1.28	6.26	7457	46.1
**2**	5.23	9.25	1.20	6.25	7339	53.2
**3**	5.28	9.27	1.26	6.26	7322	58.9
**4**	5.29	9.43	1.14	6.24	7320	54.3
**5**	5.28	9.50	1.09	6.20	7320	53.2
**6**	4.15	9.51	1.19	6.23	7314	57,6
**7**	5.28	9.51	1.12	6.16	7309	65.4
**8**	5.27	9.51	1.13	6.13	7314	56.3
**9**	5.27	9.51	1.09	6.11	7311	57.1
**10**	4.03	9.51	1.09	6.12	7307	57.8
**11**	5.27	9.51	1.12	6.07	7308	58.1

Physical and chemical parameters measured in situ in the water column of Inca Coya Lake, Antofagasta Region, Chile.

### Lithology and correlation of sediment cores

**Fig 2:**
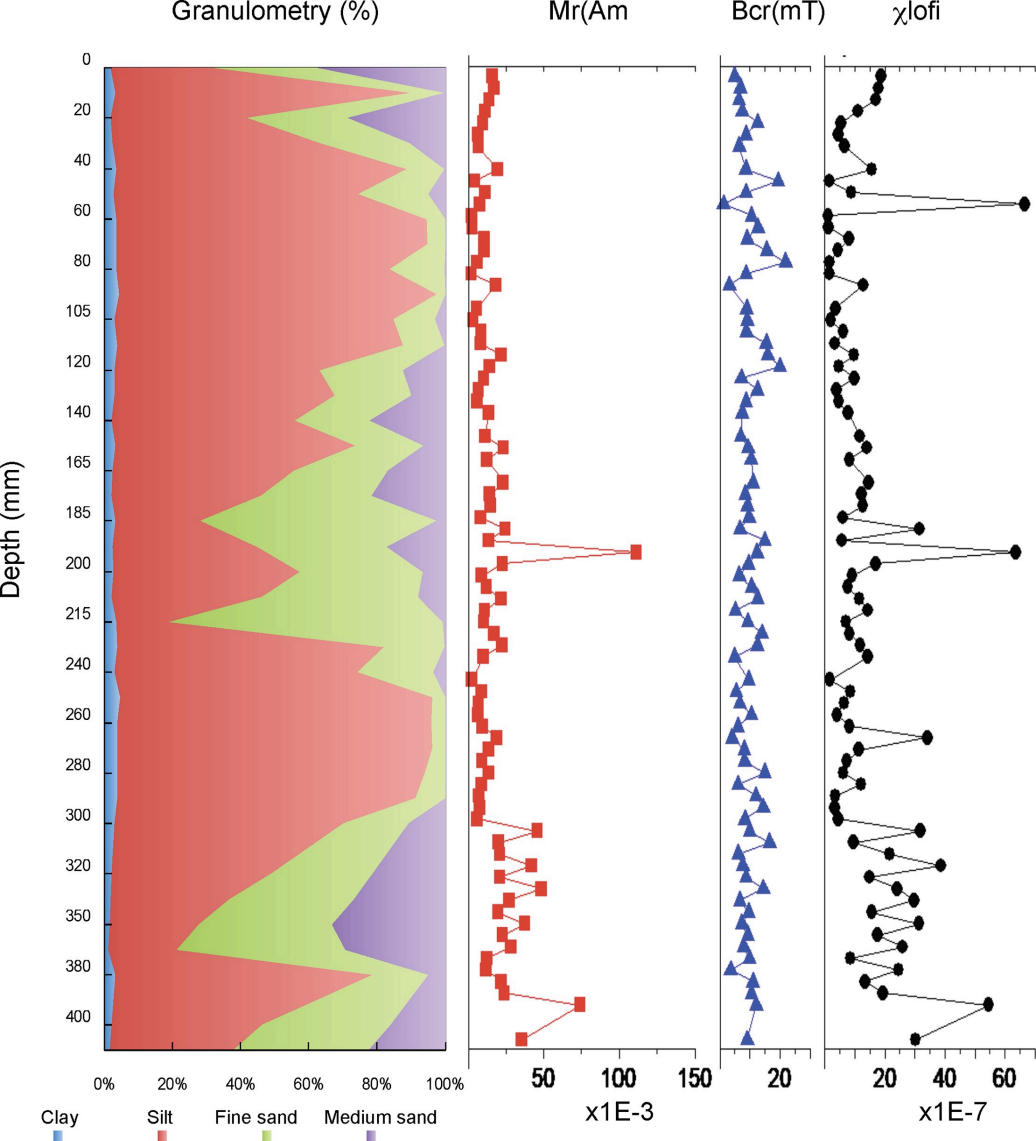
Analysis of CORE1. Grain size, magnetic properties χlofi: low-field susceptibility, χhifi: high-field susceptibility, Ms: saturation magnetization, Mr: remnant magnetization, Hc: coercive force, and Hcr: remnant coercive force).

The lithology of CORE1 and CORE2 is characterized by laminated facies of sandy silt (light brown color) with brown massive silt (*gyttja*). The fine-grained sediments In CORE1 are interrupted by three detrital episodes between 370mm and 300mm (episode D3), between 200mm and 120mm (episode D2), and in the uppermost 50mm of the sequence (episode D1), which have relatively high sand content (Fig 2). The sandy intervals of episodes D3, D2, and D1 consist of fine (0.35–0.25 mm average particle size) to medium sand (0.5–0.35 mm average particle size). The intervals exhibit a fining-upward pattern with coarser grains at the bottom and a gradual fining towards the top. According to the results of grain size analysis, sand content of episodes D3 and D2 reached up to 80% at the bottom, whereas in episode D1 sand contents did not exceed 60%.

The chronological framework for CORE1 spanned a period between 2013 (year of sampling) to approximately 1886 AD. The grey-scale data from CORE 1 and CORE 2 were used to correlate both sediment cores (S2 Fig). Four layers were interpreted as tie-points (named 1, 2 3 and 4 in S2 Fig) for the cross correlation between the cores; they correspond to abrupt decreases in the grey-scale values identified in both cores. Statistical correlation (evaluated through correlation coefficient values) between cores was significantly improved when tie-points were matched: R^2^ = 0.50 around the tie-point 1 (samples number = 21), R^2^ = 0.70 around the tie-point 2 (samples number = 10), R^2^ = 0.64 around the tie-point 3 (samples number = 8), and R^2^ = 0.61 around the tie-point 4 (samples number = 11), suggesting that the selected tie-points in CORE1 and CORE2 correspond to similar and simultaneous trends in the grey-scale values of the sediment cores.

According to the ^210^Pb dating of CORE1 [27], cross-correlation allowed the determination of the chronological framework for CORE2. Therefore, the age-depth model was obtained for CORE2 (S3 Fig). Chronological data show that CORE2 is associated with an elapsed time from 2015 AD to 1949 AD. Geochemical data were also used to corroborate the cross-correlation between CORE1 and CORE2 (S2 Fig). The four tie-points also control the main fluctuations in the metal concentrations (mainly for As and Cu). In conclusion, the grey-scale values and the metals concentrations from the sediment cores allowed the correlation of the ^210^Pb dating of CORE1 and CORE2.

### Grain size and magnetic properties

Grain-size distribution and magnetic properties for CORE1 are shown in Fig 2. Similar behavior in the calculated hysteresis curves was observed in all samples, which exhibit mainly a strong ferromagnetic behavior accompanied by a variable diamagnetic behavior. Some samples also exhibited a weak paramagnetic behavior, explained by the shape of the curves, the high low-field susceptibility (χlofi) and magnetization (Ms and Mr) values, and negative (or low) high-field susceptibility (χhifi) values. The samples are characterized by medium coercive force (Hc) and remanent coercive force (Hcr) values (see S1 Fig).

The Ms values varied from 7.72x10^-1^ to 1.37 x10^-2^ (Am^2^ kg^-1^) with an average value of ca. 1.59x10^-1^ (Am^2^ kg^-1^). The Ms values were relatively homogeneous, with only one isolate peak. The Ms curve pattern is similar to χlofi and ferrimagnetic susceptibility (χferri) (S1 Fig), which indicates that the curve is controlled mainly by the concentration of ferromagnetic minerals, more specifically ferrimagnetic minerals (e.g. [47, 66]). The Mr values varied between 1.11x10^-1^ and 1.62 x10^-3^ (Am^2^ kg^-1^) with an average of 1.66x10^-2^ (Am^2^ kg^-1^) and exhibit a curve pattern similar to Ms. These high relatively Mr values together with medium coercive values and Hc and Hcr average values of ca. 10 and 60 (mT), respectively, indicate that the ferromagnetic phase has the potential to record a significant amount of magnetization when subjected to a high magnetic field (1000 mT), which depends on the composition of the dominant magnetic phase and/or the magnetic grain size (e.g. [47, 66]). The highest susceptibility (χlofi and χferri) and magnetization (Ms and Mr) values in the Inca Coya Lake sediment core were measured in subsamples with a high amount of sand (Fig 2).

Thermomagnetic experiment results showed a reversible behavior for all samples analyzed from ~330° to ~710°C, whereas below 330°C an irreversible behavior was observed (S1 Fig). This is probably linked to a mineral phase transformation during heating in a non-inert atmosphere (oxygen) (e.g., [67]). Similar behavior is observed in all curves: a) an increase in the curves between approximately 25° and 320°C, probably associated with the presence of metastable oxyhydroxides, such as ferrihydrite (e.g., [45]) followed by b) a drastic change in the slope of the curve at ca. 330°C suggesting the presence of Fe-sulfide. After this abrupt change in the slope, the susceptibility showed a progressive decrease from ~ 340 to 580°C, probably associated with the presence of a magnetic oxidized phase, likely minerals belonging to the titanomagnetite group. Only samples Chiu03 (1.5 cm depth) and Chiu40 (20 cm depth) had a progressive decrease in the susceptibility values from ~ 580° to 680°C, which indicates the presence of a more oxidized magnetic phase (titano-maghemite/hematite). Analyses of the normalized gradient of thermomagnetic curves [51] confirmed the presence of Fe-sulfide, probably greigite, (Tc1) and nearly pure magnetite (Tc2) in all samples (S1 Fig). This observation suggests that the magnetic signal is mainly controlled by the dominance of greigite rather than Fe-Ti oxides, which in turn would indicate reducing instead of oxic conditions at the bottom of the lake.

### Geochemical analysis

**Fig 3:**
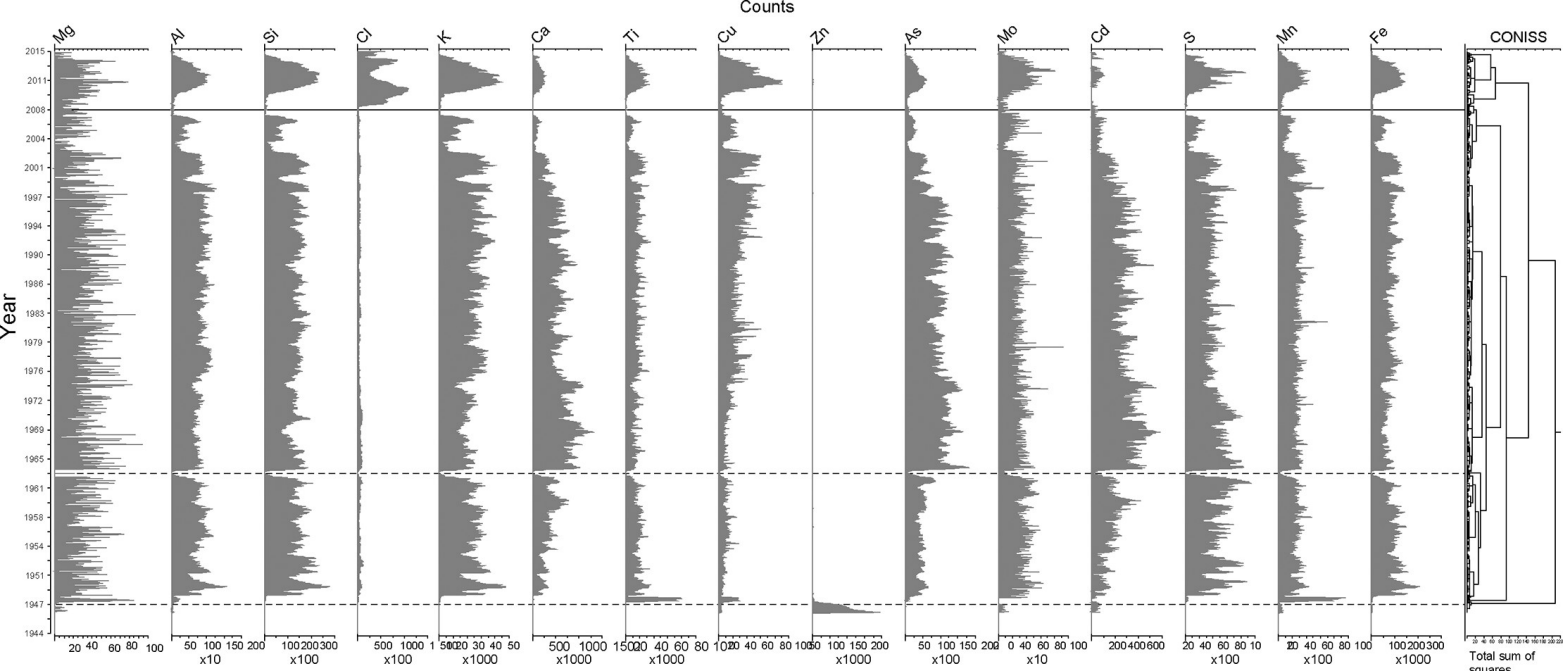
Geochemical analysis of CORE2. Elemental composition of sediment core CORE2 from Inca Coya Lake by XRF analysis and environmental periods identified by CONISS analysis.

The geochemical signature in CORE2 (Fig 3) had fluctuations in the major elements and metal(loid)s that correlate with the main lithological fluctuations of the lacustrine record, episodes D1, D2 and D3. The cluster analysis for the geochemical profiles identifies similar zones to those obtained for the grain size and magnetic properties, suggesting a strong relationship between these parameters.

The geochemical results of CORE2 exhibit distinct fluctuations correlated with the main changes in lithology. The uppermost interval (episode D1) had an abrupt increase in almost all the geochemical parameters, especially Cl, Al, Si, K, Cu, Mo, S, Mn and Fe. The pattern of episode D1 correlates well with the same interval detected in CORE1 and is linked to the high sand content in D1 (Figs 2 and 3). Episode D2 is also detected as an increase in several geochemical parameters, Ca, As, and Cd. Although the increase in their concentrations is not as abrupt as in episode D1, cluster analysis clearly separates this event.

### Biological proxies

**Fig 4:**
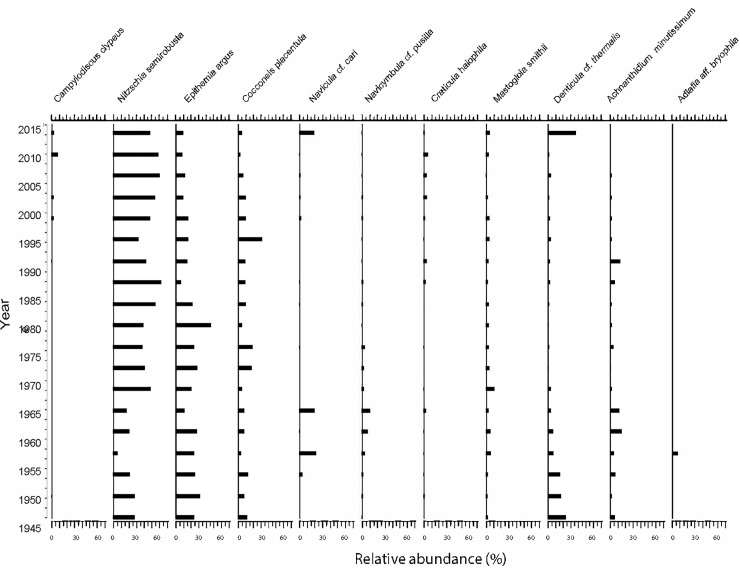
Diatom assemblage. Diatoms species and their abundance identified from Inca Coya core.

Diatom composition is shown in Fig 4. Some species are present throughout the core, some of them in high abundances (*Nitzschia semirobusta*, *Epithemia argus* and *Cocconeis placentula*) and others with very low density (*Mastoglia smithii* and *Acanthidium minutissimum*). Other species, *Navicula* cf. *cari* and *Denticula* cf. *thermalis*, occurred in the deeper samples, disappeared at intermedium depth and re-appeared in the surface sediment. The diatom assemblage included a higher frequency of benthic rather than planktonic diatoms, with clear dominance of species characteristics of oligotrophic environments.

**Fig 5:**
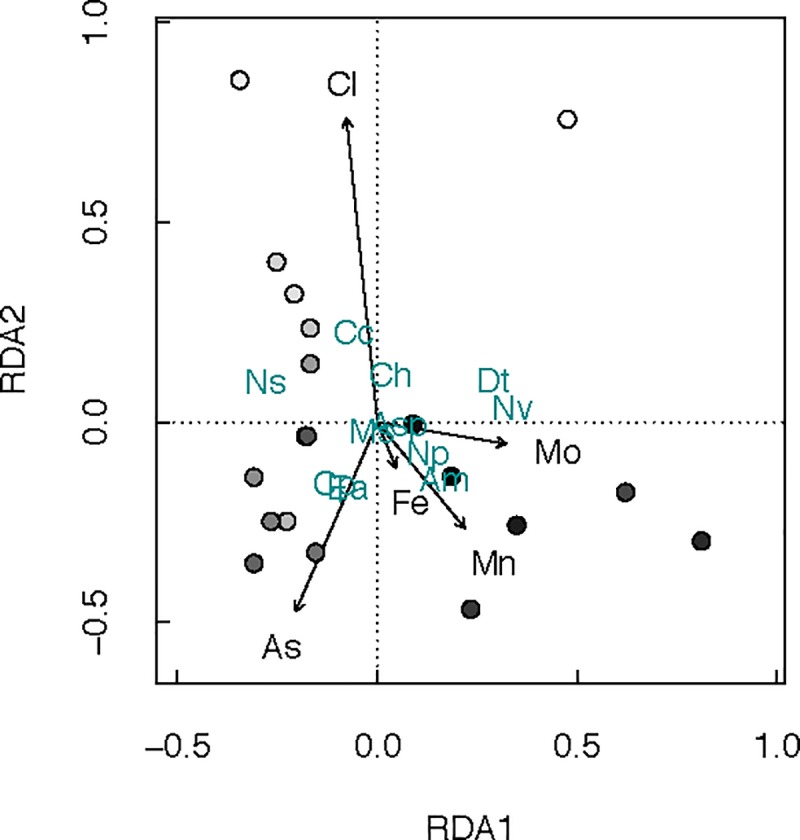
Redundancy analysis. Plot of redundancy analysis (RDA) ordination (axes 1 and 2) for 19 samples with diatom data. The arrows represent the significant environmental variables explaining variation in the diatom assemblages. Species names are in cyan colour and symbolized by initials (Cc: *Campylodiscus clypeus*; Ns: *Nitzschia semirobusta*; Ea: *Epithemia argus*; Cp: *Cocconeis placentula*; Nv: *Navicula veneta*; Np: *Navicymbula cf*. *Pusilla*; Ch: *Craticula halophila*; Ms: *Mastogloia smithii*; Am: *Achnanthidium cf*. *Minutissimum*; Asp: Achnanthes sp.; Dt: *Denticula cf*. *thermalis)*. The points represent depth of samples in the core: white points are top core samples and bottom core samples in black.

RDA ordination was used to analyze and verify the influence of geochemical and magnetic susceptibility variables on the changes in the diatom assemblages (Fig 5). Permutations and forward selection identified Cl, As, Fe, Mn and Mo as significant environmental variables for RDA ordination analysis (p< 0.05, n = 26). The first two RDA axes were statistically significant, accounting for 50.8% and 24.6% of the explained variance, respectively. Three main groups could be identified. A first cluster of species positively correlated with peaks in Cl identified in the top sediment (*Campylodyscus clypeus*, *N*. *semirobusta*, *Craticula halophila*). A second group is associated with high As concentrations at intermediate depth (*C*. *placentula* and *E*. *argus*) and a third group is correlated with the bottom sediment with elevated concentrations of Fe, Mn and Mo (*A*. *minutissimum*, *Navicymbula* cf. *pusilla* and *Navicula veneta*).

In the first group, *C*. *clypeus* is commonly found in shallow (meso)-haline inland saline lakes dominated by carbonate and sulfate [68]. *N*. *semirobusta*, the most abundant species found in CORE2, is a species more frequently found in oligo-mesotrophic conditions [69]. In the second group, *C*. *placentula*, is an indicator of moderate to good water quality. The species is epiphytic and grows in benthic habitats, where it adheres to rocks, macrophytes and algae. It is common in brackish and freshwater bodies and is widely distributed, particularly where the pH is neutral or alkaline. It is representative of shallow lakes. In third group, *A*. *minutissimum* is one of the most frequent benthic diatoms of freshwater worldwide [70]. This species has been linked to alkaline and acidic, mainly oligotrophic and mesotrophic waters and can be considered highly tolerant to metal contamination.

**Fig 6:**
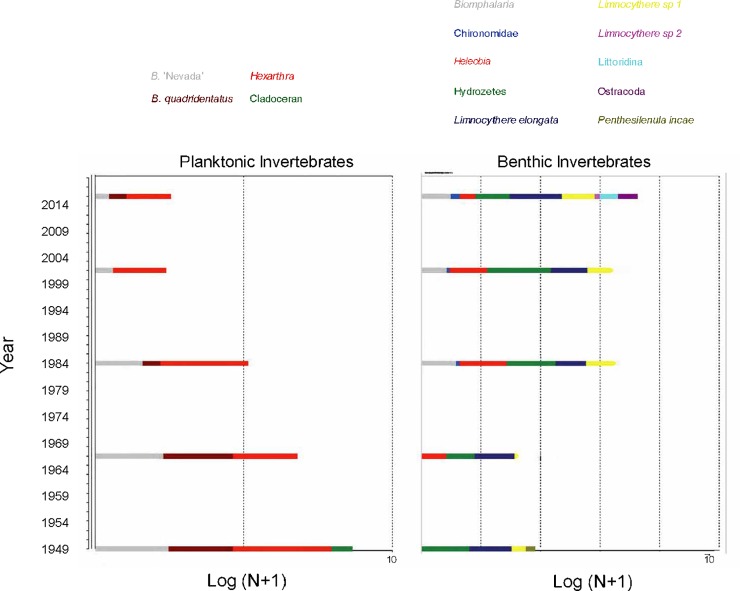
Planktonic and benthic abundance in sediment record. Planktonic (diapausing eggs in the eggs bank) and benthic invertebrates and their abundance identified from Inca Coya core.

There was an inverse pattern between diversity of benthic invertebrates and plankton egg banks (Fig 6). Higher plankton diversity (promoted by higher richness and abundances) was observed at the bottom of the core and decreased to the surface sediments. Conversely, diversity of benthic invertebrates increased from bottom to top of the sediment record.

**Fig 7:**
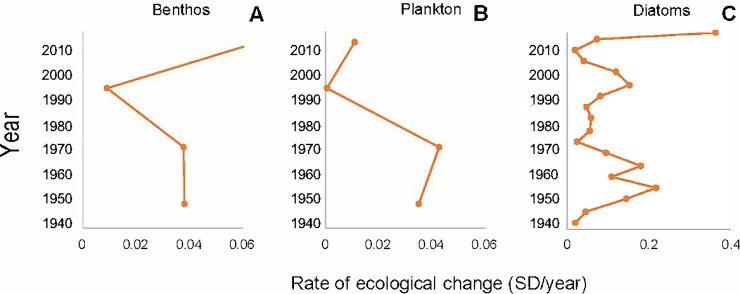
Rates of change of bioindicators. Euclidean distance between contiguous samples in terms of their ordination on the DCA rescaled ecological space; units are standard deviation (SD) as a metric for rate of change (RoC) calculated for the Inca Coya record for (a) benthic invertebrates, (b) plankton (diapausing eggs in the egg bank) and (c) diatoms identified in Inca Coya core.

The rate of change in benthic and planktonic invertebrates reached similar values from the bottom to 10 cm depth. For plankton egg banks this result is promoted by the presence of ephippia of cladocerans in the deepest sampled sediments (15–20 cm). In more recent sediments ephippia disappeared from the record. A decrease of RoC around 5 cm depth marks the beginning of recovery of the benthos. The plankton community recorded by diapausing egg banks, however, remained reduced, which could be explained by the permanence of the same rotifer species (Fig 7A and 7B). For diatoms, RoC suggests that higher diatom species turnover occurred at 11.5–13.5 cm depth, 4.5 cm depth and surface (0.5 cm depth) sediments (Fig 7C).

## Discussion

Our results showed evidence of laminated facies in the lacustrine record in Inca Coya Lake, which are interpreted as varves preserved in the bottom of the lake. Varves are associated with oxygen-depleted conditions in the bottom waters of lacustrine systems, since variation in varve preservation is a proxy for hypolimnetic hypoxia oscillations [71–72]. Past environmental changes in the lacustrine system are indicated by the observed variations between laminated and massive intervals in Inca Coya Lake sediment that are also observed in CORE1 [27], and by the presence of detrital episodes D1, D2 and D3. These lithological marker horizons as well as the magnetic parameters, Xlofi, X ferri, Ms and Mr, were correlated with episodes of wetter conditions in the lake catchment. Wetter conditions may be attributed to an increasing flood frequency during summer rains, when a hydrological and hydrogeological connection between the drainage line and the lake is inferred. The past floods have great scientific implications for the Atacama Desert. These events generate debris flows, caused by great magnitude pluvial events [73]. The fining-upwards trend of each detrital episode confirms a flood-derived deposition of the sandy horizons at the bottom of the lake. Flooding and erosion associated with extreme wet conditions has been recently recorded, e.g., in the 2001 episode of the Atacama flood, driven by exogenous precipitation during a La Niña event [19]. Conversely, under drier conditions a disconnection between the drainage line and Inca Coya Lake triggers hypoxic conditions at the bottom of the lacustrine system. However, the DO in Inca Coya Lake shows a well-oxygenated water column without oxycline. Nevertheless, this record did not reach the bottom of the lake and a decrease of DO at the water-sediment interface is quite likely, as there are expected changes in the redox potential around the interface zone of lakes where the oxygen consumption rate is high [74].

Examination of possible environmental drivers of these hypoxia periods suggests that hypolimnetic hypoxia oscillations in Inca Coya Lake might be related to alternating wetter and drier episodes in the catchment area of the Loa River. These climate variables could have influenced hypolimnetic hypoxia, likely causing changes in the intensity of watershed erosion and water mixing during flash floods as a result of the summer rains (i.e. the annual wet period associated with sporadic and extreme rainfall in the arid area of northern Chile). Preservation in varves correlates with the detrital episodes, suggesting that flooding events are the main factor controlling the distribution of varves in the lacustrine record.

Sedimentation rate follows approximately the trend of the accumulated Southern Oscillation Index (SOI) from the period 1876–2010 [27]. Specifically, the sedimentation rate during the 20^th^ century increased steadily from the 1970s, reaching a maximum at the beginning of the 1990s followed by a decrease until the year 2000. The sedimentation rate was quite stable up to approximately 1945 and then it increased until the 1980s, a period dominated by La Niña events, then decreased until the 1990s when El Niño was dominant. This suggests a potential relationship of ENSO phenomena and the sedimentary regime in the northern Chilean desert environment, at least over the last approximately 150 years. Studies conducted in the Huasco Valley (Atacama Region) also suggest that the stream flow of the Huasco River and the piezometric levels of the alluvial aquifer are strongly controlled by the ENSO signal [75]. Thus several water resources of northern Chile (lacustrine systems, streamflow from rivers and water levels of aquifers) provide evidence of this relationship. Similar effects have been recorded at northern edge of Atacama Desert in Andean valleys of Peru's western slope, Moquegua Valley, where flood hazards have been linked to ENSO variability [33].

Hydrological data from the DGA (Chilean Water Agency) in the watershed of Inca Coya Lake also shows the relationship between flash floods and the detrital episodes in the lacustrine record of the lake. According to the streamflow record of Loa River near the lake between the period 1971 and 2017, maximum and catastrophic values of streamflow were detected in 2012, 2001 and 1975 [76]. Records of total streamflows were 11.28 m^3^·s^-1^ for the year 2012, 25.51 m^3^·s^-1^ for 2001, 3.54 m^3^·s^-1^ for the 1975. All these records were in periods January-February, associated with the summer rainfalls. For the rest of years of the elapsed time (1971–2017), streamflow was mainly below 1 m^3^·s^-1^. According to the chronological model obtained from the dating and the correlation of CORE 1and CORE 2, episode D1 was deposited by the floods of 2012 and 2001, and episode D2 was deposited in the 1975 event. Episode D3 corresponds to a pre-1950 AD deposition with no historical record from DGA.

The magnetic signal in Inca Coya sediments is dominated mainly by Fe-sulfides (Tc1) and to a lower extend by titanomagnetite (Tc2). Fe-sulfides are interpreted to be of authigenic origin, since significant sources of Fe-sulphide in the watershed are missing. We therefore suggest that Fe-sulfides were formed by transformation of Fe-Ti oxides of detrital origin in a reducing environment [77]. It is suggested that Fe-sulfides contained in the sediments likely originated from authigenic formation of greigites (Curie temperatures ~330°C) in reducing environments. Fe-Ti oxides in the sediments consist of nearly-pure magnetite (Curie temperatures ~580°C) that are derived from the erosion of the existing rocks in the catchment area (e.g., volcanic, plutonic and sedimentary rocks), which contain significant amounts of these minerals [66, 78]. The highest susceptibility (χlofi and χferry) and magnetization (Ms and Mr) values coincide with higher sand content in the lake sediments, which corresponds to horizons with higher concentration of Fe-bearing minerals [47]. This correlation could be a consequence of a higher detrital input under humid climatic condition (pluvial events, [67]). The episodic pluvial events would cause greater oxygenation of the bottom water, which is reflected in the presence of more oxidized magnetic minerals (titano-maghemite/hematite) in the sand samples. In summary, both, laminated facies observed and magnetic signals in the lacustrine record were consistent with the temporality of the floods and the detrital episodes occurred in the region during the analyzed period, with consequences in oxygenation conditions of Inca Coya sediments.

Our results suggest the significant role of the Pacific Ocean as a source of humidity in a wide latitudinal range in the Atacama Desert. The hydrological and hydrogeological behavior of such a broad area is firmly explained by the water provided by the ENSO climatic fluctuations during recent times, the last decades and the last centuries, providing a useful climatic information associated with the water recharge in northern Chile. Lakes, rivers, aquifers and springs located on the western slope of the Andean Range are mainly recharged by the humidity from the Pacific Ocean, and this ENSO linkage allows the understanding of the mechanism that triggers the water recharge. Water availability for inhabitants in northern Chile strongly depends on the pattern of streamflow, groundwater levels, springs and lake levels, which can be understood in terms of the ENSO variability. From this point of view, Inca Coya Lake is sensitive to the global climatic changes and does not respond only to local environmental factors related to the catchment area. Hence, the Inca Coya lacustrine system provides a representative short-term paleoenvironmental reconstruction of Atacama Desert at 22°S and it can be correlated to paleoenvironmental records located between 17°S and 28°S.

Bioavailability of compounds in aquatic ecosystems can be highly dependent on other physicochemical parameters (e.g. pH, DOC, etc.). Studies have shown that low pH, in combination with moderately oxygenated redox conditions increases the bioavailability of metals. Therefore aquatic organisms that are subjected to acidification bioconcentrate more metals, which may be transferred to higher trophic levels by grazing [79]. Iron (Fe) plays an integral role in many biological processes such as photosynthesis, respiration, processing of reactive oxygen species and nutrient acquisition [80]. Iron input and bioavailability in aquatic environments have far-reaching repercussions for many natural systems. Fe acquisition by means of reduction is a widespread Fe uptake strategy in aquatic systems. Although magnetic susceptibility cannot significantly explain the diatoms community distribution from Inca Coya, Fe and Mn were significant variables, which are among the characteristic inorganic oxidants in water bodies [80] and are commonly related to redox conditions in these environments.

*A*. *minutissimum* was recorded in the diatom assemblages identified from Inca Coya sediment, it is considered an opportunistic species, highly tolerant to metal contamination. The species dominates polluted lakes in mining areas and positively correlates with Cu and Zn [81, 82]. Although *A*. *minutissimum* was not the most abundant species in Inca Coya sediments, it was present throughout the entire core. *C*. *placentula* is an epiphytic species that is found in littoral zones associated with plant roots. Its presence in the lowermost zone is probably associated with periods of increased runoff, identified by magnetic parameters. *C*. *placentula* and *Denticula* cf. *thermalis* have also been reported in thermo-extreme environments (>45°C) [83], which suggest their broad tolerance to harsh conditions.

The egg banks in Inca Coya sediments are strongly dominated by rotifer diapausing eggs. The presence of ephippia of cladocerans in deeper sediments corresponded to ephippia containing two eggs, which disappeared in the more recent sediments and in the modern water column (personal observation). Production of diapausing eggs in many species is related to population density, with dense active populations producing more diapausing eggs as part of the bank. Our results suggest that zooplankton populations in the water column were more abundant and diverse in the past (core bottom) but decreased over time until now (i.e. during the last 90 years in the environment history in Inca Coya Lake). Zooplankton populations are frequently used to detect anthropogenic contamination, because of their sensitivity to different toxicants and their important ecosystem role. Rotifers are usually more tolerant to many toxicants than cladocerans [84]. This may suggest that environmental conditions have become unfavorable for zooplankton. Studies have assessed the effects of pollutants, especially metals, on the life cycle of rotifers, including parameters related to sexual reproduction and diapausing production and its consequent community modulator role [14, 15, 28]. Increasing concentrations of heavy metals as Cu in the Inca Coya sediments could explain this pattern, mainly affecting the more sensitive group of cladocerans, which have disappeared from the lake. In a previous study, we tested experimentally the toxic effect of Cu on the rotifer *Brachionus* ‘Nevada’ isolated from Inca Coya Lake. As expected, Cu has a negative effect on different demographic parameters of the life cycle of the rotifer species, but we detected great tolerance of individuals hatched from the diapausing egg bank, which suggests local adaptation of the Inca Coya population to the presence of Cu [85], and selection of tolerant populations of more sensitive rotifer species [28].

Our results also suggest that invertebrate community structure (primary consumers) reflected metal exposure better than diatom composition (primary producers), measured as changes in assemblage composition through species turnover. Diatom community composition was best associated with variables related to wetter/drier alternation and consequent changes in oxygen availability, which promote oxidizing or reducing environments. Although As was a significant chemical variable explaining diatom distribution over time, it is not possible to discount its natural presence in the Atacama Desert basins and therefore to attribute these concentrations to anthropic contamination only. In fact, the Salado River is As-rich due to water from the El Tatio geothermal fields, which reaches levels up to 30 mg L^-1^ As [86, 87] and the extremely arid conditions and high evaporation maintain high concentrations of the metalloid throughout the Loa River’s course [88, 89]. The As profile did not follow the same behavior as Cu, more directly associated with anthropic contamination, which suggests a natural origin of at least part of the As concentration in Inca Coya sediments. Similar conclusions were reported by Hirst et al. [90], who studied polluted rivers; macroinvertebrate diversity, richness and total abundance declined and evenness increased with increasing metal concentrations, but among diatoms, pH and conductivity explained the main variations in assemblage composition, and neither diversity, richness nor evenness varied with metal concentration. Due to its genesis from the dissolution of carbonaceous rocks, Inca Coya Lake is a typical Ca-rich lake. The invertebrates present in the region have important adaptations to environmental conditions of salinity and alkalinity and some minerals and metals, as has been observed in invertebrates from other karstic systems [91]. Our results suggest that both factors, climate and metal(loid)s exposure would act as modulators of aquatic community structure, where alternation of wetter/drier events and their consequent changes in oxygen availability make a bottom-up control and the differential sensitivity to metal(loids) exposure would impact on higher trophic levels of aquatic community.

There is broad consensus about the sensitivity of lakes to climate, showing that physical, chemical, and biological lake properties respond rapidly to climate-related changes [92]. Lakes are therefore considered ‘sentinels’ of current climate change [93] but lake sediments also contain valuable historical archives of both long- and short-term natural and anthropogenic environmental changes, which interact in a dynamical way along time. Thus, biological proxies are useful and widely used to identify environmental periods in decadal scales. Diatom community composition varies with changes in the physical and chemical conditions of lakes, as shown in our results, making them excellent water quality indicators [94, 95], more specifically of lake trophic status, eutrophication and organic pollution. Including groups of higher trophic levels such as filterers, grazers or predators in paleoecological assessments, which have different sensitivities to different pressures, allow identifying the impact of multiple stressors as past selective forces that modify the ecosystems that will face future environmental conditions. Paleohydrological reconstruction of the study area constitutes a useful tool to understand the spatial and temporal variability of ENSO in this circum-Pacific area of the Southern Hemisphere and led to the identification of water recharge episodes in northern Chile during the last centuries. Information about temporal variation of humid conditions and floods events in arid zones, as we showed for Inca Coya Lake, should help to guide the sustainable use of its water resources and the conservation efforts of natural aquatic systems, not only facing critical climatic scenarios, but also the increasing pollution and high water demand for increasing human consumption and industrial use.

## Conclusions

Multi-proxy analysis of the recent sedimentary record of Inca Coya Lake has demonstrated the presence of wetter and drier periods during the last 90 years. The lake, which is located very close to the Salado River, reflected detailed periods which have been attributed to alternate episodes of hydrological/hydrogeological connection and disconnection with the drainage line of the watershed. Paleolimnological criteria (lithology, grain size, magnetic susceptibility and geochemistry) identified three detrital episodes associated with the increase of flash flood events in the catchment area of Loa River. In particular, limnological criteria (species of diatoms, diapausing egg bank and invertebrate community) were demonstrated to be excellent indicators of the bioavailability of compounds in the aquatic ecosystem of Inca Coya Lake. Fluctuations in their species discerned environmental episodes triggered by natural processes (e.g. the existence of intense rainfall events during the summer) compared to anthropic processes (e.g. the mining activity located near the lacustrine system). Therefore, the aquatic system constitutes an excellent sentinel of recent environmental changes in the Loa River catchment area, allowing the environmental impact assessment on the water resources due to flash floods or mining activity in the driest desert of the world.

## Supporting information

S1 Fig Curie temperatures. Curie temperatures (Tc) of subsamples of Inca Coya Lake sediments. (A) Chiu03 (1.5 cm depth), (B) Chiu09 (4.5 cm depth), (C) Chiu26 (13 cm depth), and (D) Chiu40 (20 cm depth). In red lines heating curve and blue lines cooling curve.
(PDF)

S2 Fig Correlation between CORE1 and CORE2. Correlation between CORE1 and CORE2 according to the grey-scale values from the cores (profile in dark blue corresponds to CORE1, and profile in light blue is CORE2). Pb-210 dating is shown for CORE1. Four tie-points were defined as black circles named 1, 2, 3 and 4; they correspond to abrupt decreases in the grey-scale values which are identified in both sediment cores. Geochemical profiles from CORE1 (As and Cu) and from CORE2 (As, Cu and Zn) were also compared for correlation. According to the correlation of the sediment cores, the environmental episodes detected in CORE2 are attributed to historical periods from 1940 AD to the date of sampling.
(PDF)

S3 Fig Age-Depth model for CORE2. According to correlation between CORE1 and CORE2, chronological model for CORE2 is obtained.
(PDF)

## References

[1] Antonovics J, Bradshaw AD, Turner RG. Heavy metal tolerance in plants. Adv. Ecol. Res. 1971; 7, 1–85.

[2] Hamilton PB, Rolshausen G, Webster TMU, Tyler CR. Adaptive capabilities and fitness consequences associated with pollution exposure in fish. Phil. Trans. R. Soc. B. 2017; 372: 20160042 10.1098/rstb.2016.0042 27920387 PMC5182438

[3] Keller W, Yan ND, Holtze K, Pitblado JR. Chemical response of acid lakes in the Sudbury, Ontario area to reduced smelter emissions, 1981–1989. Can J Fish Aquat Sci. 1992; 49 (Suppl.1): 25–32.

[4] Pollard HG, Colbourne JK, Keller W. Reconstruction of Centuries-old *Daphnia* Communities in a Lake Recovering from Acidification and Metal Contamination. Ambio. 2003; 32: 214–218. 10.1579/0044-7447-32.3.214 12839198

[5] Korosi JB, Thienpont JR, Smol JP, Blais JM. Paleo-ecotoxicology: What Can Lake Sediments Tell Us about Ecosystem Responses to Environmental Pollutants? Environ Sci Technol. 2017; 51: 9446–9457. 10.1021/acs.est.7b02375 28763202

[6] Pigati JS, Miller DM, Bright J, Mahan SA, Nekola JC, Paces JB. Chronology, sedimentology, and microfauna of ground-water discharge deposits in the central Mojave Desert, Valley Wells, California. Geol Soc Am Bull. 2011; 123: 2224–2239.

[7] Ritter B, Wennrich V, Medialdea A, Brill D, King G, Schneiderwind S, et al Climatic fluctuations in the hyperarid core of the Atacama Desert during the past 215 ka. Sci Rep. 2019; 9: 5270 10.1038/s41598-019-41743-8 30918294 PMC6437205

[8] Hering D, Johnson RK, Kramm S, Schmutz S, Szoszkiewicz K, Verdonschot PFM. Assessment of European streams with diatoms, macrophytes, macroinvertebrates and fish: a comparative metric-based analysis of organism response to stress. Freshw Biol. 2006; 51: 1757–1785.

[9] Rogalski MA Tainted resurrection: metal pollution is linked with reduced hatching and high juveniles mortality in Daphnia egg banks. Ecology. 2015; 96: 1166–1173. 10.1890/14-1663.1 26236831

[10] Gómez A, Carvalho GR. Sex, parthenogenesis and genetic structure of rotifers microsatellite analysis of contemporary and resting egg bank populations. Mol Ecol. 2000; 9: 203–214. 10.1046/j.1365-294x.2000.00849.x 10672164

[11] Orsini L, Marshall H, Cuenca-Cambronero M, Chaturvedi A, Thomas KW, Pfrender ME, et al Temporal genetic stability in natural populations of the waterflea Daphnia magna in response to strong selection pressure. Mol Ecol. 2016; 25: 6024–6038. 10.1111/mec.13907 27862502

[12] Turko P, Sigg L, Hollender J, Spaak P. Rapid evolutionary loss of metal resistance revealed by hatching decades-old eggs. Evolution. 2016; 70: 398–407. 10.1111/evo.12859 26768308

[13] Rogalski MA. Maladaptation to Acute Metal Exposure in Resurrected Daphnia ambigua Clones after Decades of Increasing Contamination. Am Nat. 2017; 189: 443–452. 10.1086/691077 28350505

[14] Marcial HS, Hagiwara A, Snell TW. Effect of some pesticides on reproduction of rotifer *Brachionus plicatilis* Muller. Hydrobiologia. 2005; 546: 569–575.

[15] Aránguiz-Acuña A, Serra M. Metal stress in zooplankton diapause production: post-hatching response. Ecotoxicology. 2016; 26: 329–339. 10.1007/s10646-017-1766-7 28105571

[16] Quade J. Paleowetlands and regional climate change in the central Atacama Desert, northern Chile. Quaternary Res. 2008; 69(3): 343–360.

[17] Jordan T, Kirk-Lawlor NE, Blanco N, Rech J, Cosentino N. Landscape modification in response to repeated onset of hyperarid paleoclimate states since 14 Ma, Atacama Desert, Chile. Geol Soc Am Bull. 2014; 126 (7/8): 1016–1046.

[18] Cerveny R. Present climates of South America In: Hobbs J.E. (Ed.), Climates of the Southern Continents: Present, Past and Future. John Wiley and Sons, Chichester, UK; 1998 pp. 107–135.

[19] Houston J. Variability of precipitation in the Atacama Desert: Its causes and hydrological impact. Int J Climatol. 2006; 26: 2181–2198.

[20] Abbott MB, Wolfe BB, Wolfe AP, Seltzer GO, Aravena R, Mark BG, et al Holocene paleohydrology and glacial history of the central Andes using multiproxy lake sediment studies. Palaeogeogr Palaeocl. 2003; 194: 123–138.

[21] Sillitoe RH. Porphyry copper systems. Econ Geol. 2010; 105(1): 3–41.

[22] Tapia J, Murray J, Ormachea M, Tirado N, Nordstrom DK. Origin, distribution, and geochemistry of arsenic in the Altiplano-Puna plateau of Argentina, Bolivia, Chile, and Perú. Sci Total Environ. 2019; 678: 309–325. 10.1016/j.scitotenv.2019.04.084 31075598

[23] COCHILCO. Proyección de la producción de cobre en Chile 2018–2029. DEPP 15/2018. 2018.

[24] Dittmar T. Hydrochemical process controlling arsenic and heavy metal contamination in the Elqui river system (Chile). Sci Total Environ. 2004; 325: 193–207. 10.1016/j.scitotenv.2003.11.005 15144789

[25] Salvarredy-Aranguren MM, Probst A, Roulet M, Isaure M-P. Contamination of surface waters by mining wastes in the Milluni Valley (Cordillera Real, Bolivia): Mineralogical and hydrological influences. Appl Geochem. 2008; 23: 1299–1324.

[26] Ramírez M, Massolo S, Frache R, Correa JA. Metal speciation and environmental impact on sandy beaches due to El Salvador copper mine, Chile. Mar Pollut Bull 2005; 50, 62–72. 10.1016/j.marpolbul.2004.08.010 15664034

[27] Cerda M, Evangelista H, Valdés J, Siffedine A, Boucher H, Nogueira J, et al A new 20th century lake sedimentary record from the Atacama Desert/Chile reveals persistent PDO (Pacific Decadal Oscillation) impact. J South Am Earth Sci. 2019; 95: 102302.

[28] Aránguiz-Acuña A, Pérez-Portilla P, De la Fuente A, Fontaneto D. Life-history strategies in zooplankton promote coexistence of competitors in extreme environments with high metal content. Sci Rep. 2018; 8:11060 10.1038/s41598-018-29487-3 30038433 PMC6056428

[29] Herkovits J. Paleoecotoxicology: Extending environmental toxicology and chemistry to the interpretation of the fossil record. Environ Toxicol Chem. 2001; 20: 1623–1624. 11491541

[30] Vargas G, Ortlieb L, Rutllant J. Aluviones históricos en Antofagasta y su relación con eventos El Niño/Oscilacion del Sur. Rev Geol Chile 27 2000; (2):155–174.

[31] Jordan T, Herrera C, Godfrey LV, Colucci SJ, Gamboa C, Urrutia J, González G, Paul JF. Isotopic characteristics and paleoclimate implications of the extreme precipitation event of March 2015 in northern Chile. Andean Geol. 2019; 46: 1–31.

[32] Ortega C, Vargas G, Rojas M, Rutllant JA, Muñoz P, Lange CB, Pantoja S, Dezileau L, Ortlieb L. Extreme ENSO-driven torrential rainfalls at the southern edge of the Atacama Desert during the Late Holocene and their projection into the 21th century. Global Planet Change. 2019; 175: 226–237.

[33] Goldstein P, Magilligan FJ. Hazard, risk and agrarian adaptations in a hyperarid watershed: El Niño floods, streambank erosion, and the cultural bounds of vulnerability in the Andean Middle Horizon. Catena. 2011; 85: 155–167.

[34] Lamy F, Hebbeln D, Wefer G. Late Quaternary precessional cycles of terrigenous sediment input off the Norte Chico, Chile (27.5°S) and palaeoclimatic implications. Palaeogeogr Palaeocl. 1998; 141(3–4): 233–251.

[35] Lamy F, Klump J, Hebbeln D, Wefer G. Late Quaternary rapid climate change in northern Chile. Terra Nova. 2000; 12: 8–13.

[36] Rojas M, Moreno PI, Kageyama M, Crucifix M, Hewitt C, Abe-Ouchi A, et al The Southern Westerlies during the Last Glacial Maximum in PMIP2 simulations. Clim Dynam. 2009; 32(4): 525–548.

[37] Stuut J-BW, Lamy F. Climate variability at the southern boundaries of the Namib (southwestern Africa) and Atacama (northern Chile) coastal deserts during the last 120,000 yr. Quaternary Res. 2004; 62(3): 301–309.

[38] Díaz FP, Latorre C, Maldonado A, Quade J, Betancourt JL. Rodent middens reveal episodic, long-distance plant colonizations across the hyperarid Atacama Desert over the last 34,000 years. J Biogeogr. 2012; 39: 510–525.

[39] Maldonado A, Betancourt JL, Latorre C, Villagrán C. Pollen analyses from a 50,000-yr rodent midden series in the southern Atacama Desert (25 degrees 30'S). J Quaternary Sci. 2005; 25 20(5): 493–507.

[40] Betancourt JL, Latorre C, Rech JA, Quade J, Rylander KA. A 22,000-Year Record of Monsoonal Precipitation from Northern Chile's Atacama Desert. Science. 2000; 289 (5484): 1542–1546. 10.1126/science.289.5484.1542 10968788

[41] Bobst AL, Lowenstein TK, Jordan TE, Godfrey LV, Ku T-L, Luo S. A 106 ka paleoclimate record from drill core of the Salar de Atacama, northern Chile. Palaeogeogr Palaeocl. 2001; 173(1–2): 21–42.

[42] Gayo EM, Latorre C, Jordan TE, Nester PL, Estay SA, Ojeda KF, Santoro C. Late Quaternary hydrological and ecological changes in the hyperarid core of the northern Atacama Desert (similar to 21 degrees S). Earth-Sci Rev. 2012; 113(3–4): 120–140.

[43] Diederich JL, Wennrich V, Bao R, Büttner C, Bolten A, Brill D, et al A 68 ka precipitation record from the hyperarid core of the Atacama Desert in northern Chile. Global Planet Change. 2020; 103054.

[44] Naranjo JA, Paskoff RP. Estratigrafía de los depósitos Cenozoicos de la Región Chiu Chiu-Calama, Desierto de Atacama. Revista Geológica de Chile. 1981; 13–14: 81–83.

[45] Ziervogel K, Bohling B. Sedimentological parameters and erosion behaviour of submarine coastal sediments in the south-western Baltic Sea. Geo-Mar Lett. 2003; 23: 43–52.

[46] Smith KA. Soil and Environmental Analysis: Physical Methods, Revised, and Expanded. Taylor & Francis 2000.

[47] Evans ME, Heller F. Environmental magnetism: principles and applications of enviromagnetics (Vol. 86). Academic press 2003.

[48] Tauxe L. Paleomagnetic principles and practice. Modern approaches in geophysics Kluwer Academic Publishers 1998.

[49] Tauxe L. Essentials of Paleomagnetism. University of California Press 2005.

[50] Appleby P, Oldfieldz F. The calculation of lead-210 dates assuming a constant rate of supply of unsupported 210Pb to the sediment. Catena. 1978; 5: 1–8.

[51] Petterson G, Renberg I, Geladi P, Lindberg A, Lindgren F. Spatial uniformity of sediment accumulation in varved lake sediments in northern Sweden. J Paleolim. 1993; 9: 195–208.

[52] Krammer K, Lange-Bertalot H. Bacillariophyceae. 1. Teil: Naviculaceae In: Ettl H., Gerloff J., Heynig H. and Mollenhauer D. (eds.) Süsswasserflora von Mitteleuropa, Band 2/1. Gustav Fisher Verlag, Jena; 1986. 876 pp.

[53] Bennett KD. Determination of the number of zones in a biostratigraphical sequence. New Phytol. 1996; 132(1): 155–170. 10.1111/j.1469-8137.1996.tb04521.x 33863055

[54] Legendre P, Oksanen J, ter Braak CJF. Testing the significance of canonical axes in redundancy analysis. Methods Ecol Evol. 2011; 2: 269–277.

[55] Liu Y, Chuanhong C, Yang S. Assessment of Anthropogenic Impact versus Climate Change on the Succession of the Diatom Community in Lugu Lake (Yunnan-Guizhou Plateau, China) Using the Sedimentary Record of Geochemical Elements. Water. 2019; 11: 655 10.3390/w11040655

[56] Šmilauer P, Lepš J. Multivariate Analysis of Ecological Data using CANOCO 5. Cambridge: Cambridge University Press 2014 10.1017/CBO9781139627061

[57] Blanchet FG, Legendre P, Borcard D. Forward selection of explanatory variables. Ecology. 2008; 89: 2623–2632. 10.1890/07-0986.1 18831183

[58] Legendre P, Legendre L. Numerical Ecology. 3rd English ed. Elsevier 2012.

[59] Hill MO, Gauch HG. Detrended correspondence analysis: an improved ordination technique In Classification and ordination Springer, Dordrecht 1980; pp. 47–58.

[60] Urrego DH, Bush MB, Silman MR, Correa-Metrio A, Ledru M-P, Mayle FE, et al Millennial-Scale Ecological Changes in Tropical South America Since the Last Glacial Maximum. In: Vimeux F., Sylvestre F., Khodri M. (eds) Developments in Paleoenvironmental Research Series (DPER), Springer, Paris 2009; pp. 283–300.

[61] Correa-Metrio A, Bush MB, Cabrera KR, Sully S, Brenner M, Hodell DA, et al Rapid climate change and no-analog vegetation in lowland Central America during the last 86,000 years. Quaternary Sci Rev. 2012; 38: 63–75.

[62] Oksanen J, Blanchet G, Kindt R, Legendre P, O'Hara B, Simpson GL, et al Vegan: Community Ecology Package, 1.17–3: Vienna, The R Project for Statistical Computing 2009 Available from: http://CRAN.R-project.org/package=vegan.

[63] Correa-Metrio, A., Urrego, D.H., Cabrera, K.R., Bush, M.B. PaleoMAS: Paleoecological Analysis, R package version 2.0–1 ed.:Vienna. 2011. Available from: http://CRAN.R-project.org/package=paleoMAS.

[64] OECD: Eutrophication of Waters. Monitoring, Assessment and Control.—154 pp. Paris: Organisation for Economic Co‐Operation and Development 1982.

[65] Kasprzak P, Padsák J, Koschel R, Krienitz L, Gervais F. Chlorophyll a concentration across a trophic gradient of lakes: An estimator of phytoplankton biomass? Limnologica. 2008; 38: 327–338.

[66] Dunlop DJ, Özdemir Ö. Rock magnetism: fundamentals and frontiers (Vol. 3). Cambridge university press 2001.

[67] Pizarro H, Rousse S, Riquelme R, Veloso E, Campos E, González R, et al The origin of the magnetic record in Eocene-Miocene coarse-grained sediments deposited in hyper-arid/arid conditions: Examples from the Atacama Desert. Palaeogeogr Palaeocl. 2019; 516: 322–335.

[68] Poulícbreve;ková A, Jahn R. *Campylodiscus clypeus* (Ehrenberg) Ehrenberg ex Kützing: typification, morphology and distribution. Diatom Res. 2007; 22(1): 135–146.

[69] Rodrigues-Bartozek EC, Zorzal-Almeida S, Bicudo DC. Surface sediment and phytoplankton diatoms across a trophic gradient in tropical reservoirs: new records for Brazil and São Paulo State. Hoehnea. 2008; 45(1): 69–92.

[70] Krammer K, Lange-Bertalot H. Bacillariophyceae. 3. Teil: Centrales, Fragilariaceae, Eunotiaceae In Ettl H., Gerloff J., Heynig H. & Mollenhauer D. (Eds.). Süsswasserflora von Mitteleuropa. Gustav Fisher Verlag, Stuttgart, Germany 1991; pp. 1–576.

[71] Salminen S, Saarni S, Tammelin M, Fukumoto Y, Saarinen T. Varve Distribution Reveals Spatiotemporal Hypolimnetic Hypoxia Oscillations During the Past 200 Years in Lake Lehmilampi, Eastern Finland. Quaternary. 2019; 2(2): 20.

[72] Zolitschka B, Francus P, Ojala AE, Schimmelmann A. Varves in lake sediments—A review. Quaternary Sci Rev. 2015; 117: 1–41.

[73] Grosjean M, Nuñez L, Cartajena I, Messerli B. Mid-holocene climate and culture change in the Atacama Desert, northern Chile. Quat Res. 2017; 48(2): 239–246.

[74] DeLaune RD, Reddy KR. Redox Potential In: (Hillel D ed) Encyclopedia of Soils in the Environment. Academic Press 2005; pp. 366–371.

[75] Salas I, Herrera C, Luque JA, Delgado J, Urrutia J, Jordan T. Recent climatic events controlling the hydrological and the aquifer dynamics at arid areas: The case of Huasco River watershed, northern Chile. Sci Total Environ. 2016; 571: 178–194. 10.1016/j.scitotenv.2016.07.132 27471983

[76] Dirección General de Aguas. Ministerio de Obras Públicas, Chile. [cited 14 November 2019]. Available from: https://dga.mop.gob.cl.

[77] Roberts AP, Reynolds RL, Verosub KL, Adam DP. Environmental magnetic implications of greigite (Fe3S4) formation in a 3 my lake sediment record from Butte Valley, northern California. Geophys Res Lett. 1996; 23(20): 2859–2862.

[78] Sáez A, Cabrera L, Garcés M, van den Bogaard P, Jensen A, Gimeno D. The stratigraphic record of changing hyperaridity in the Atacama Desert over the last 10 Ma. Earth Planet. Sci Lett. 2012; 355: 32–38.

[79] Tao Y, Yuan Z, Xiaona H, Wei M. Distribution and bioaccumulation of heavy metals in aquatic organisms of different trophic levels and potential health risk assessment from Taihu lake, China. Ecotox Environ Safe. 2011; 81:55–64. 10.1016/j.ecoenv.2012.04.014 22633085

[80] Shaked Y, Lis H. Disassembling iron availability to phytoplankton. Front Microbiol. 2012; 3 10.3389/fmicb.2012.00123 22529839 PMC3328120

[81] Cantonatti M, Angeli N, Virtanen L, Wojtal AZ, Gabrieli J, Falasco E, et al *Achnanthidium minutissimum* (Bacillariophyta) valve deformities as indicators of metal enrichment in diverse widely-distributed freshwater habitats. Sci Total Environ. 2014; 475: 201–215. 10.1016/j.scitotenv.2013.10.018 24377680

[82] Tolloti R, Consani S, Carbone C, Vagge G, Capello M, Cutroneo L. Benthic diatom community response to metal contamination from an abandoned Cu mine: Case study of the Gromolo Torrent (Italy). J Environ Sci. 2019; 75: 233–246. 10.1016/j.jes.2018.03.034 30473289

[83] López-Sandoval O. Diversidad algal de un ambiente extremo: el manantial geothermal Los Hervideros, México. Rev Mex Biodivers. 2016; 87(1). 10.1016/j.rmb.2016.01.004

[84] Rico-Martínez R, Arzate-Cárdenas MA, Robles-Vargas D, Pérez-Legaspi IA, Alvarado-Flores J, Santos-Medrano GE. Rotifers as Models in Toxicity Screening of Chemicals and Environmental Samples. IntechOpen. 2015; 10.5772/61771

[85] Aránguiz-Acuña A, Pérez-Portilla P. Metal stress in zooplankton diapause production: post-hatching response. Ecotoxicology. 2017; 26: 329–339. 10.1007/s10646-017-1766-7 28105571

[86] Romero L, Alonso H, Campano P, Fanfani L, Cidu R, Dadea C, et al Arsenic enrichment in waters and sediments of the Río Loa (Second Region, Chile). Appl Geochem. 2003; 18: 1399–1416.

[87] DGA. Diagnóstico y clasificación de los cursos y cuerpos de agua según objetivos de calidad Cuenca río Loa. Gobierno de Chile. Ministerio de Obras Públicas 2004.

[88] Pell A, Márquez A, López-Sánchez JF, Rubio R, Barbero M, et al Ocurrence of arsenic species in algae and freshwater plants of an extreme arid region in northern Chile, the Loa River Basin. Chemosphere. 2013; 90: 556–564. 10.1016/j.chemosphere.2012.08.028 22981629

[89] Pizarro I, Gómez-Gómez M, León J, Román D., Palacios MA. Bioaccessibility and arsenic speciation in carrots, beets and quinoa from a contaminated area of Chile. Sci Total Environ. 2016; 565: 557–563. 10.1016/j.scitotenv.2016.04.199 27196992

[90] Hirst H, Jüttner I, Ormerod SJ. Comparing the responses of diatoms and macroinvertebrates to metals in upland streams of Wales and Cornwall. Freshw Biol. 2002; 47: 1752–1765.

[91] Ariani AP, Wittmann KJ. The transition from an Epigean to a Hypogean Mode of Life: Morphological and bionomical characteristics of Diamysis camassai sp.nov. (Mysidacea, Mysidae) from brackish-water dolinas in Apulia, SE-Italy. Crustaceana. 2001; 74: 1241–1265.

[92] Rosenzweig C. Assessment of observed changes and responses in natural and managed systems In: Parry ML, Canziani OF, Palutikof JP, van der Linden PJ, Hanson CE, et al (eds). Climate change 2007—impacts, adaptation and vulnerability. Contribution of Working Group II to the Fourth Assessment Report of the Intergovernmental Panel on Climate Change. Cambridge Univ. Press 2007; pp. 79–131.

[93] Adrian R, O’Reilly CM, Zagarese H, Baines SB, Hessen DO, Keller W, et al Lakes as sentinels of climate change. Limnol Oceanogr. 2009; 54(6): 2283–2297. 10.4319/lo.2009.54.6_part_2.2283 20396409 PMC2854826

[94] Bellinger EG, Sigee DC. Freshwater Algae: Identification and Use as Bioindicators. John Wiley & Sons, Ltd 2010.

[95] Guilizzoni P, Lami A, Marchetto A, Appleby PG, Alvisi F. Fourteen years of palaeolimnological research of a past industrial polluted lake (L. Orta, Northern Italy): an overview. J Limnol. 2001; 60: 249–262.

