# Supplementary material for: Aquatic community structure as sentinel of recent environmental changes unraveled from lake sedimentary records from the Atacama Desert, Chile
Source: PLoS One. 2020 Feb 21;15(2):e0229453. doi: 10.1371/journal.pone.0229453 (PMC7034912; doi:10.1371/journal.pone.0229453)
Supplement: S1 Fig — Curie temperatures (Tc) of subsamples of Inca Coya Lake sediments. (A) Chiu03 (1.5 cm depth), (B) Chiu09 (4.5 cm depth), (C) Chiu26 (13 cm depth), and (D) Chiu40 (20 cm depth). In red lines heating curve and blue lines cooling curve. (PDF) [file pone.0229453.s001.pdf]

Supporting information: Appendix S1. Adriana Aránguiz-Acuña, José A. Luque, Héctor Pizarro, Mauricio Cerda, Inger Heine-Fuster, Jorge Valdés, Emma Fernández-Galego, Volker Wennrich

Aquatic community structure as sentinel of recent environmental changes unraveled from sedimentary records from an Atacama Desert Lake, Chile

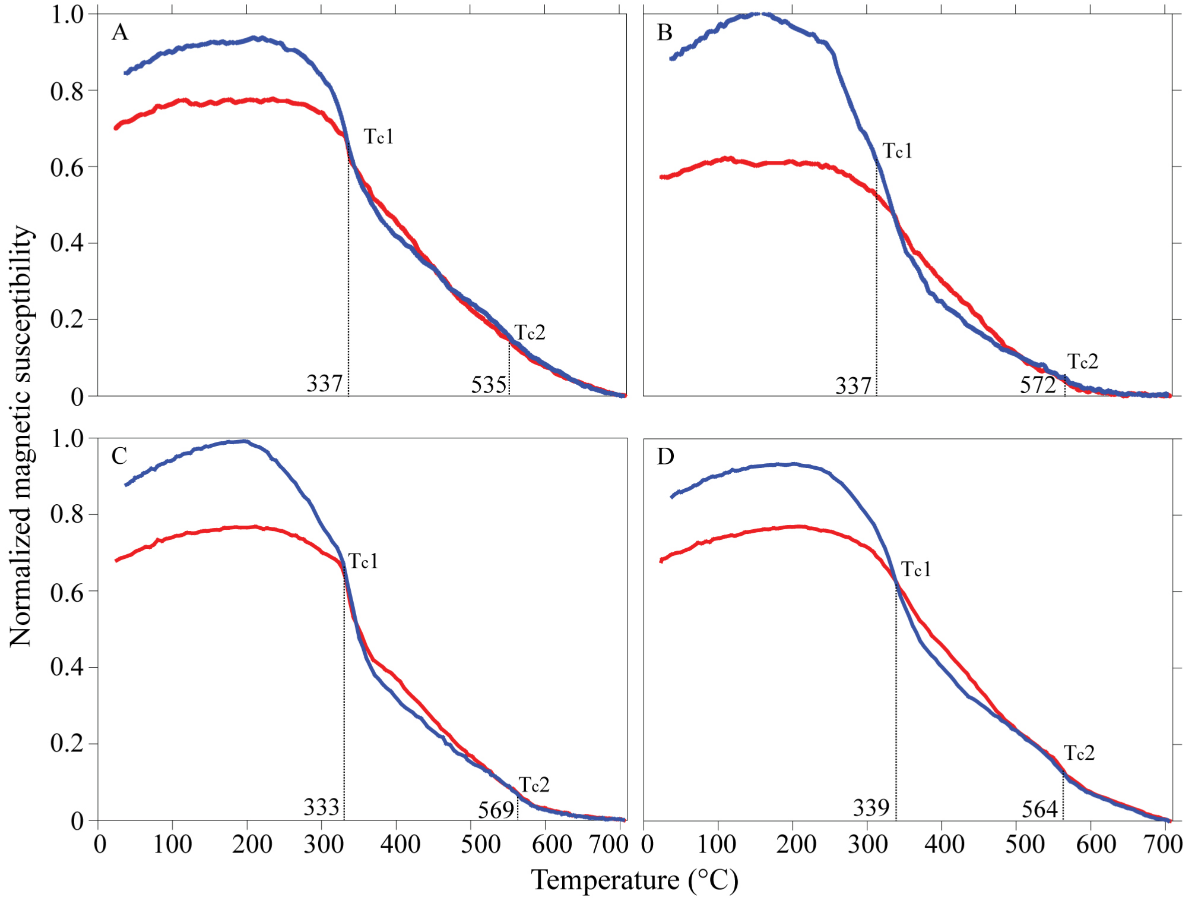

S1 Fig. Curie temperatures. Curie temperatures (Tc) of subsamples of Inca Coya Lake sediments. (A) Chiu03 (1.5 cm depth), (B) Chiu09 (4.5 cm depth), (C) Chiu26 (13 cm depth), and (D) Chiu40 (20 cm depth). In red lines heating curve and blue lines cooling curve.
