# Supplementary material for: Aquatic community structure as sentinel of recent environmental changes unraveled from lake sedimentary records from the Atacama Desert, Chile
Source: PLoS One. 2020 Feb 21;15(2):e0229453. doi: 10.1371/journal.pone.0229453 (PMC7034912; doi:10.1371/journal.pone.0229453)
Supplement: S2 Fig — Correlation between CORE1 and CORE2 according to the grey-scale values from the cores (profile in dark blue corresponds to CORE1, and profile in light blue is CORE2). Pb-210 dating is shown for CORE1. Four tie-points were defined as black circles named 1, 2, 3 and 4; they correspond to abrupt decreases in the grey-scale values which are identified in both sediment cores. Geochemical profiles from CORE1 (As and Cu) and from CORE2 (As, Cu and Zn) were also compared for correlation. According to the correlation of the sediment cores, the environmental episodes detected in CORE2 are attributed to historical periods from 1940 AD to the date of sampling. (PDF) [file pone.0229453.s002.pdf]

Supporting information: Appendix S2. Adriana Aránguiz-Acuña, José A. Luque, Héctor Pizarro, Mauricio Cerda, Inger Heine-Fuster, Jorge Valdés, Emma Fernández-Galego, Volker Wennrich

Aquatic community structure as sentinel of recent environmental changes unraveled from sedimentary records from an Atacama Desert Lake, Chile

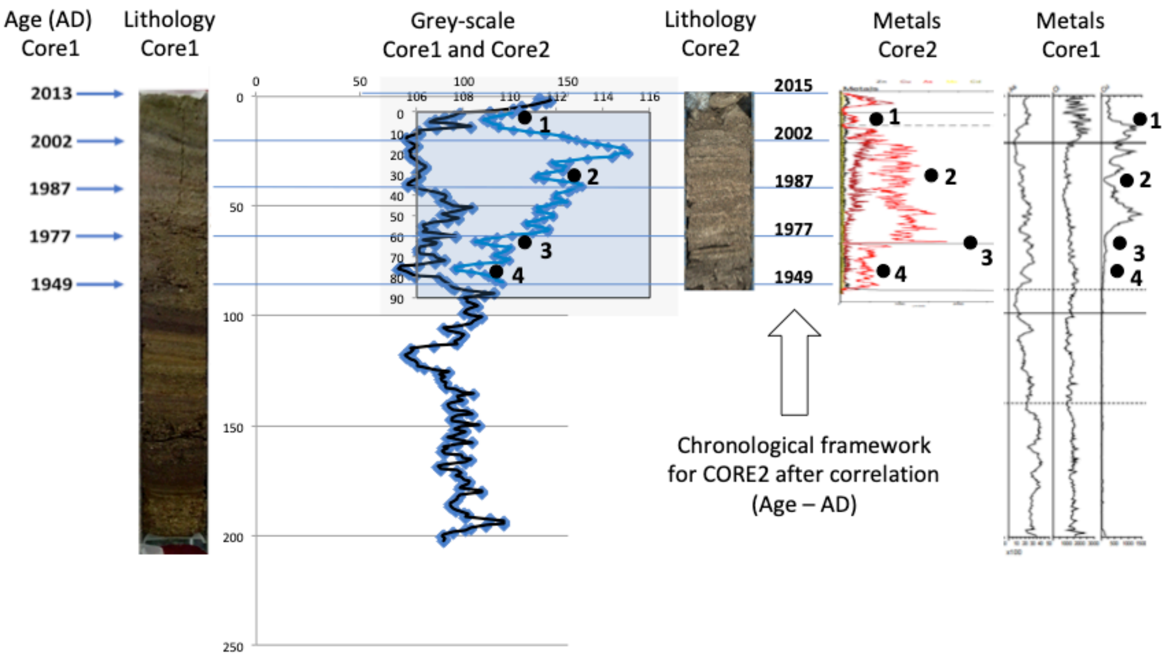

S2 Fig. Correlation between CORE1 and CORE2. Correlation between CORE1 and CORE2 according to the grey-scale values from the cores (profile in dark blue corresponds to CORE1, and profile in light blue is CORE2). Pb-210 dating is shown for CORE1. Four tie-points were defined as black circles named 1, 2, 3 and 4; they correspond to abrupt decreases in the grey-scale values which are identified in both sediment cores. Geochemical profiles from CORE1 (As and Cu) and from CORE2 (As, Cu and Zn) were also compared for correlation. According to the correlation of the sediment cores, the environmental episodes detected in CORE2 are attributed to historical periods from 1940 AD to the date of sampling.
