# Supplementary material for: Aquatic community structure as sentinel of recent environmental changes unraveled from lake sedimentary records from the Atacama Desert, Chile
Source: PLoS One. 2020 Feb 21;15(2):e0229453. doi: 10.1371/journal.pone.0229453 (PMC7034912; doi:10.1371/journal.pone.0229453)
Supplement: S3 Fig — According to correlation between CORE1 and CORE2, chronological model for CORE2 is obtained. (PDF) [file pone.0229453.s003.pdf]

Supporting information: Appendix S3. Adriana Aránguiz-Acuña, José A. Luque, Héctor Pizarro, Mauricio Cerda, Inger Heine-Fuster, Jorge Valdés, Emma Fernández-Galego, Volker Wennrich

Aquatic community structure as sentinel of recent environmental changes unraveled from sedimentary records from an Atacama Desert Lake, Chile

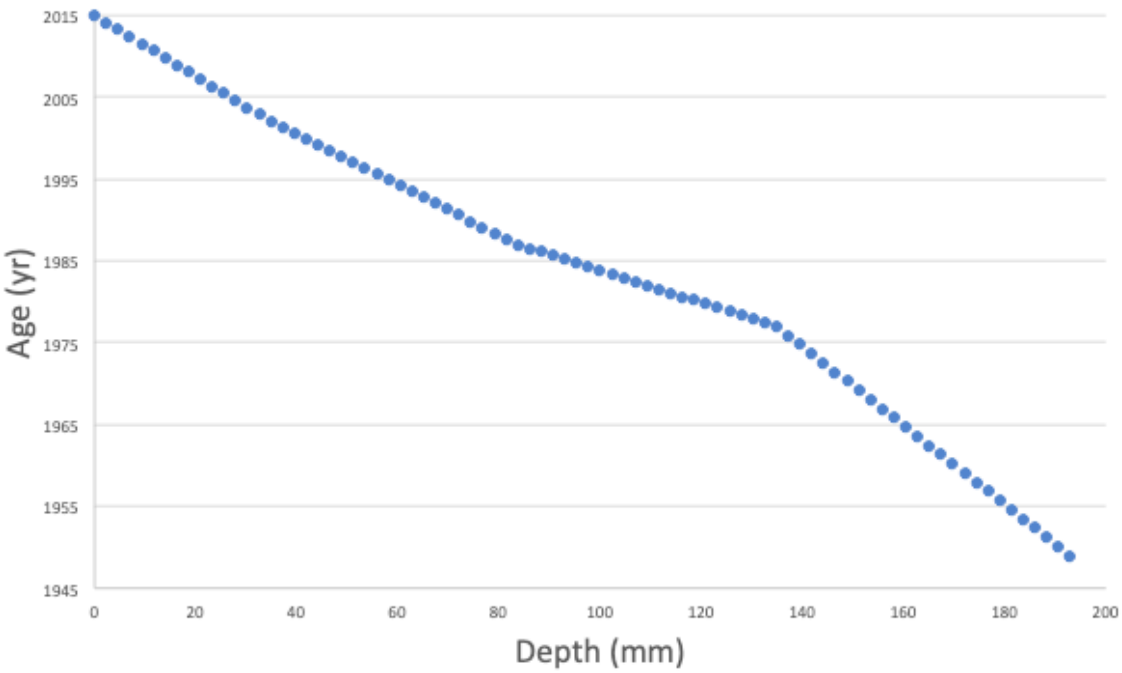

S3 Fig. Age-Depth model for CORE2. According to correlation between CORE1 and CORE2, chronological model for CORE2 is obtained.
